# Discriminating between the Effects of Founding Events and Reproductive Mode on the Genetic Structure of *Triops* Populations (Branchiopoda: Notostraca)

**DOI:** 10.1371/journal.pone.0097473

**Published:** 2014-05-13

**Authors:** Rebekah L. Horn, Ralph Kuehn, Victoria Drechsel, David E. Cowley

**Affiliations:** 1 Department of Fish, Wildlife & Conservation Ecology and Molecular Biology Program, New Mexico State University, Las Cruces, New Mexico, United States of America; 2 Unit of Molecular Zoology, Chair of Zoology, Department of Animal Science, Technische Universität München, Freising, Germany; University of Massachusetts, United States of America

## Abstract

Crustaceans that initially colonize a freshwater temporary pond can strongly bias the subsequent genetic composition of the population, causing nearby populations to be genetically distinct. In addition, these crustaceans have various reproductive modes that can influence genetic differentiation and diversity within and between populations. We report on two species of tadpole shrimp, *Triops newberryi* and *Triops longicaudatus* “short”, with different reproductive modes. Reproduction in the tadpole shrimp can occur clonally (parthenogenesis), with self fertilization (hermaphroditism), or through outcrossing of hermaphrodites with males (androdioecy). For all these reproductive modes, population genetic theory predicts decreased genetic diversity and increased population differentiation. Here we use mitochondrial control region (mtCR) sequences and nuclear microsatellite loci to determine if the difference in reproductive mode affects the high genetic structure typical of persistent founder effects. Previous authors indicated that *T. newberryi* is androdioecious because populations are composed of hermaphrodites and males, and *T. longicaudatus* “short” is hermaphroditic or parthenogenetic because males are absent. In our data, *T. newberryi* and *T. longicaudatus* “short” populations were highly structured genetically over short geographic distances for mtCR sequences and microsatellite loci (*T. newberryi*: Φ_ST_ = 0.644, *F*_ST_ = 0.252, respectively; *T. l.* “short”: invariant mtCR sequences, *F*_ST_ = 0.600). Differences between the two *Triops* species in a number of diversity measures were generally consistent with expectations from population genetic theory regarding reproductive mode; however, three of four comparisons were not statistically significant. We conclude the high genetic differentiation between populations is likely due to founder effects and results suggest both species are composed of selfing hermaphrodites with some level of outcrossing; the presence of males in *T. newberryi* does not appreciably reduce inbreeding. We cannot exclude the possibility that males in *T. newberryi* are non-reproductive individuals and the two species have the same mating system.

## Introduction

Aquatic invertebrates that disperse passively via an encysted embryo use a variety of transport methods to colonize new habitats. Abiotic factors, such as water and wind, [1], [2], [3], [4], [5] and biotic vectors, such as birds, mammals, insects, amphibians and human activity can disperse invertebrates large distances [3], [6], [7], [8], [9], [10], [11], [12], [13], [14]. Colonization of new habitats by a combination of these factors can be relatively quick, especially if ponds are located in close proximity [1], [2]. The potential for dispersal, however, does not always equate to the actual immigration into ponds that is occurring by aquatic invertebrates [15]. It is commonly observed that populations of many aquatic invertebrates can have a high degree of genetic differentiation despite being located in close proximity [16], [17], [18], a result not expected if contemporary dispersal is frequently occurring between populations.

In cyclically parthenogenetic zooplankton, De Meester et al. [19] emphasized the importance of local adaptation for monopolizing resources, thereby creating genetic differentiation between ponds in close proximity. Boileau et al. [20] concluded that founder events, not contemporary gene flow, have a pronounced effect on the population genetic structure of aquatic invertebrates that produce resting eggs. To demonstrate that genetic “barriers” are formed to inhibit immigration into populations, Boileau et al. [20] used simulations to show that *F*_ST_ does not decay for at least 2000 generations in a large population established by a few founders and subsequently experiencing migrant influx.

In addition to founder events, the mode of reproduction can also influence the amount of genetic structure and diversity in large Branchiopods [21]. A population with individuals that reproduce via selfing experience a heterozygote deficit and decreased diversity due to small effective population sizes [22], [23]. In addition, compared to species that outcross, populations of selfing individuals are genetically more isolated because of limited gene flow and often experience demographic fluctuations [22], [23].

The tadpole shrimp (*Triops* sp.) is a passively dispersing aquatic crustacean that has been said to use several forms of reproductive modes including parthenogenesis, hermaphroditism, androdioecy (a mix between outcrossing and hermaphrodites) and gonochorism (males and females that outcross) [24], [25], [26]. Within *Triops* populations, low genetic diversity, deviations from Hardy-Weinberg equilibrium, large inbreeding values (*F*_IS_) and large population differentiation have been observed and has been attributed to founder events and the degree of outcrossing between individuals [18], [21], [27], [28], [29], [30].

Many of the previous studies have focused on *Triops* populations that are separated by distances of hundreds or thousands of kilometers between sampled ponds [21], [27], [31]. Large geographic distances between populations makes it difficult to determine if it is the mating system influencing the genetic structure and diversity of *Triops* populations or if dispersal of encysted embryos is simply limited over long distances. The current study is designed to aid in differentiating between the influence of founding events, dispersal and mating systems by using nine *Triops* populations located within 30 km and encompassing two putative species with different presumed reproductive modes. Two of the species of *Triops* in the northern Chihuahuan Desert are *T. longicaudatus* “short” and *T. newberryi* [27], [32]. Different reproductive modes are presumed for *T. l*. “short” and *T. newberryi* based on the male (absence of a brood pouch) to female (presence of a brood pouch) ratio within populations; *T. l*. “short” is comprised of all females and is assumed to reproduce via parthenogenesis or hermaphroditism whereas *T. newberryi* is thought to be androdioecious, with populations comprised of hermaphrodites that outcross with males [27]. A recent phylogeny of *Triops* showed that *T. l*. “short” and *T. newberryi* are not monophyletic, calling into question whether species status is warranted [33].

The first objective of this study is to assess the genetic structure of each *Triops* species and determine what factors (founding events or contemporary dispersal) influence population differentiation. Secondly, we compare the effect of different presumed reproductive modes and the degree of inbreeding to the genetic diversity and structure of the *Triops* populations. We hypothesize, based on population genetic theory, that the androdioecious species will have more alleles, higher allelic richness, fewer private alleles, higher observed heterozygosity, lower *F*_IS_ and *F*_ST_, and relatively greater genetic variance within as opposed to between populations. The last objective is to evaluate whether the two putative species of *Triops* in southern New Mexico are reproductively isolated in the ponds in which they co-occur.

## Materials and Methods

### Sampling Methods and Study Sites

**Figure 1:**
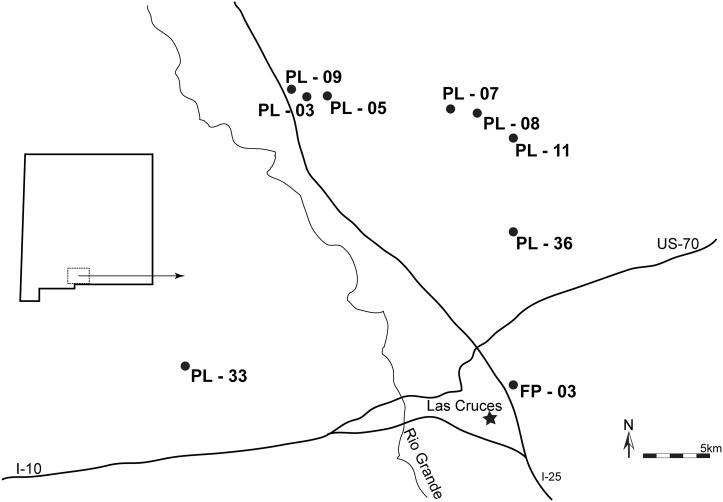
Locations of sampled playas within New Mexico, USA (inset) in relation to Las Cruces, NM. The playa lakes PL-08, PL-09, and PL-33 contain only *T. l*. “short”; PL-11, PL-36, and FP-03 contain only *T. newberryi*; and PL-03, PL-05, and PL-07 contain both species.

There were no special permits required to sample *Triops* as they are not listed as a protected species. *Triops newberryi* and *T. l*. “short” samples were collected in 2008–2011 at nine temporary ponds in southern New Mexico on public land, the Chihuahuan Desert Rangeland Research Center (CDRRC) and the Jornada Experimental Range (JER), both owned by New Mexico State University: two natural playa lakes (PL-07, PL-09), six modified playa lakes (PL-03, PL-05, PL-08, PL-11, PL-33, PL-36), and one man-made flood retention pond (FP-03) (GPS coordinates: Table S1 in File S1). All ponds were located near Las Cruces, New Mexico, USA within the Chihuahuan Desert (Fig. 1). The two species co-occurred at three of the playa lakes (PL-03, PL-05 and PL-07) (Fig. 1). Live samples were collected using 3 mm mesh seines and immediately placed in 95% ethanol for preservation. A description of field sampling methods, sample locations, and morphological verification of species, are given by Macdonald et al. [32]. Each specimen was evaluated for the presence or absence of a brood pouch to determine sex of the individual. Samples were stored at −20°C until DNA isolation.

### Mitochondrial Control Region Sequencing

Extraction of DNA followed a modified version of the HotShot method described by Montero-Pau et al. [34]. Aliquots of 75 µl of the lysis buffer and neutralizing solution were added and samples were incubated at 95°C for 45 minutes. Amplification of the mitochondrial control region (mtCR) was performed by polymerase chain reaction (PCR) with newly developed primers (dloopF 5′GCACGAGTTAAGCCGATCTT; dloopR 5′CCACATGATTTACCCTATCAAGG) for *T. newberryi* (n = 160) and *T. l*. “short” (n = 66). Reaction volumes of 25 µl consisted of 10 µl GoTAQ Green Master Mix (Promega Corp., Madison, WI), 400 pM of each forward and reverse primer and 1 ng/µl of genomic DNA. PCR reactions were run in a Fisher thermocycler (Fisher Scientific Inc., Pittsburgh, PA) with the following conditions: 94°C for 2 minutes, followed by 35 cycles of 94°C for one minute denaturation, 50°C for one minute annealing, 72°C for one minute elongation and a final extension of 72°C for 15 minutes. PCR products were checked for strength of amplification on a 1% agarose gel. Purification of PCR products was performed with ExoSAP-IT (USB Corporation, Clevland, OH) following manufacturer’s protocol. Purified PCR products were then sequenced by NMSU’s MOLBIO Molecular Analysis Service in both forward and reverse directions (http://mmas.research.nmsu.edu).

Sequences were aligned using the assembly function in the program Geneious Pro v5.4.6 [36]. Summary statistics, which included number of haplotypes, number of substitutions, number of transitions/transversions, number of polymorphic sites, nucleotide diversity (*π*), and haplotype diversity (*h*), for each playa and all samples collectively were obtained by Arlequin v3.5 [37]. Relationships between haplotypes (maternal lineages) were resolved using the program TCS [38] to construct a haplotype network at the 95% confidence level.

The appropriate substitution model (TrN+I) for the data was selected based on the Akaike information criterion (AIC) using Modeltest v3.7 [39]. The genetic distance between populations and between haplotypes using the p-distance and the Tamura-Nei model were calculated in the program MEGA v5.05 with uniform rates among sites and gaps treated as missing data [40].

The program Arlequin v3.5 [37] was used to calculate an AMOVA and also generate pairwise Φ_ST_ values. A sequential Bonferroni analysis [41] was utilized to correct for multiple, nominal tests in the pairwise analysis. Pairwise genetic distances [Φ_ST_/(1−Φ_ST_)] were directly compared to log transformed pairwise geographic distance between the playas to test an isolation by distance hypothesis with the IBDWS v3.21 utility on the web that uses a Mantel test for the analysis [42], [43].

### Microsatellite Genotyping

Genomic DNA for the microsatellite analysis was extracted by the phenol-chloroform protocol [35] and DNA was stored at −20°C. A total of 163 *T. newberryi* from six ponds and 156 *T. l*. “short” samples from six ponds were genotyped for eight loci developed specifically for *Triops* species found in southern New Mexico (TL-L-1, TL-S-5, TL-S-9, TL-S-13, TN-6, TN-7, TN-13, TN-14 [30]) and one microsatellite designed for *T. cancriformis* (TCB-99 [21]). To guarantee the loci are informative for both species [44], loci were chosen based on the primers’ ability to PCR-amplify DNA in both species and secondly to contain more than one allele per locus in order to avoid ascertainment bias as described in Ellegren et al. [45]. PCR reactions were done in an UNO II cycler (Biometra, Göttingen) in a 15 µl reaction volume containing 0.3 pmol/µl forward and reverse primers (biomers.net, Ulm), 0.1 pmol/µl Cy5-labled M13 [46], 1X PCR-Buffer (10x reaction buffer without detergent or MgCl_2_; BD Solis Biodyne, Tartu, Estonia), 2.5 mM MgCl_2_, 0.2 mM DNTPs, 0.04 U/µl Taq-Polymerase (Fire Pol DNA polymerase, Solis Biodyne, Tartu, Estonia) and 2 ng/µl genomic DNA. Microsatellite PCR reaction conditions were 95°C for 3 minutes, followed by 35 cycles of 94°C for 30 seconds denaturation, primer specific annealing temperature for 60 seconds, and 72°C for 60 seconds elongation, before a final extension at 72°C for 3 minutes. Genotyping of all samples was performed on an Automated Laser Flourescence (ALF) II express (Amersham Pharmacia Biotech, Nürnbrecht). Internal and external standards as well as one reference sample (previously sequenced sample) were included on each gel to facilitate consistent scoring across gels. Alleles were scored with the AlleleLinks 1.02 software (Amersham Parmacia Biotech).

The expected and observed heterozygosity of each population and linkage disequilibrium were calculated in Genepop on the Web v4.0.10 [47] and Bonferroni corrections were applied [41]. The dataset was checked for null alleles using the program FreeNA [48] and applying the EM algorithm [49] to compare amplification success of non-specific microsatellite markers on the different species. Values are not reported due to the difficulty of assessing heterozygote deficit vs. null alleles in organisms with high inbreeding [50] and are instead used only as a proxy for successful marker amplification. GenAlEx v6.41 [51] was used for an AMOVA to determine amount of variation between and within populations. The overall and pairwise *F*_ST_, their significance values, *F*_IS_ (the inbreeding coefficient), and allelic richness was calculated in FSTAT v2.9.3.2 as well as a two-sided statistical test to compare allelic richness, observed heterozygosity, *F*_IS_ and *F*_ST_ between species [52]. The proportion of selfing (*S*) in each population was calculated, based on the estimated *F*_IS_ values, using the equation *S* = 2*F*_IS_/(1+*F*_IS_) [53]. It is noted that there can be a bias when estimating *S* if the *F*_IS_ values do not accurately represent inbreeding, and are instead from genotyping error or population substructure [50]. Estimates of inbreeding and selfing rate were compared to the percentage of males in *T. newberryi* populations, as it would be expected that an increased proportion of males would cause an increase in the rate of outcrossing, therefore lowering selfing estimates. The migration rate (*Nm*) [54] between populations of *Triops* was calculated in GenAlEx v6.41 [51]. Similar to the mitochondrial data, genetic distance, defined as *F*_ST_/(1−*F*_ST_), was compared to log geographic straight line distance between playas to detect the presence of isolation by distance using the IBDWS v3.21 utility on the web that uses a Mantel test for the analysis [42].

To visually determine the genetic structuring between populations within a species, a discriminant analysis of principal components (DAPC [55]) was performed using the adegenet v1.3–5 [56] package in the R platform v2.15.2 [57]. DAPC overlooks the within group variation and summarizes the amount of between group variation, making this method superior for assessing relationships between populations [55]. A factorial correspondence analysis (FCA) of both species together was performed in GENETIX v4.05.2 [58] to visually assess species designation and if hybrid individuals are present in the sample set [59].

## Results

### Population Structure - mitochondrial Control Region

The *T. newberryi* mtCR ranged in length from 548 to 551 base pairs long with an overall total of 24 polymorphic sites, 21 substitutions and a transition to transversion ratio of 20∶4 (Genbank accession numbers KJ627793–KJ627799). Summary statistics for each playa and for all playas combined are in Table 1.

**Table 1:** The mitochondrial control region and microsatellite summary statistics for *T. newberryi* (TN) and *T. l*. “short” (TLS) for nine temporary ponds in southern New Mexico, USA.

Population	Mit	%M	NH	*π*	*h*	NP	NS	Mst	N_A_	A_R_	P_A_	H_exp_	H_obs_	P_HW_	*F*_IS_	*S*
TN FP-03	10	28.6	4	0.014	0.533	24	21	28	3.8	3.530	2.9	0.386	0.194	*	0.501	0.667
TN PL-03*	30	13.3	2	0.001	0.067	12	11	19	2.2	2.222	10	0.206	0.023	*	0.889	0.941
TN PL-05*	30	23.3	4	0.007	0.595	19	17	30	3.0	2.785	7.4	0.354	0.093	*	0.742	0.852
TN PL-07*	30	3.3	5	0.008	0.625	13	11	26	4.7	4.358	29	0.365	0.132	*	0.642	0.782
TN PL-11	30	13.3	2	0.012	0.515	13	12	30	2.1	2.014	0	0.237	0.144	*	0.394	0.565
TN PL-36	30	26.7	1	0.000	0.000	0	0	30	3.1	2.903	11	0.235	0.111	*	0.531	0.693
TN Total*average*	160	18.0	7	0.015	0.747	28	24	163	*3.2*	*2.969*	12	*0.297*	*0.116*		0.601	*0.740*
TLS PL-03*	17	-	1	-	-	-	-	14	2.3	2.333	20	0.224	0.103	*	0.549	0.709
TLS PL-05*	10	-	1	-	-	-	-	24	2.6	2.392	8	0.269	0.093	*	0.658	0.794
TLS PL-07*	9	-	1	-	-	-	-	28	3.6	2.969	28	0.262	0.115	*	0.566	0.723
TLS PL-08	10	-	1	-	-	-	-	30	2.0	1.919	5.6	0.199	0.144	*	0.277	0.433
TLS PL-09	10	-	1	-	-	-	-	30	2.0	1.874	11	0.177	0.063	*	0.648	0.786
TLS PL-33	10	-	1	-	-	-	-	30	1.8	1.537	13	0.088	0.033	*	0.626	0.770
TLS Total*average*	66	-	1	-	-	-	-	156	*2.4*	*2.171*	16	*0.203*	*0.092*		0.547	*0.689*

The populations of *Triops* are designated as TN for *T. newberryi* and TLS for *T. longicaudatus* “short”. Table includes the number of samples sequenced for the control region (Mit) and genotyped with the microsatellites (Mst), percentage of males (%M), number of haplotypes (NH), nucleotide diversity (*π*), haplotype diversity (*h*), number of polymorphisms (NP), number of substitutions (NS), average number of alleles (N_A_), the allelic richness (A_R_), percentage of private alleles (P_A_), the expected and observed heterozygosity (H_exp_, H_obs_), probability of the Hardy Weinberg exact tests (an asterisks indicates out of HW after Bonferroni correction), the inbreeding coefficient (*F*_IS_), and the proportion of selfing (*S*) based on the *F*_IS_ value (note, the average *S* is the geometric average). The asterisks in the population column indicate that both species occur in the playa lake.

**Figure 2:**
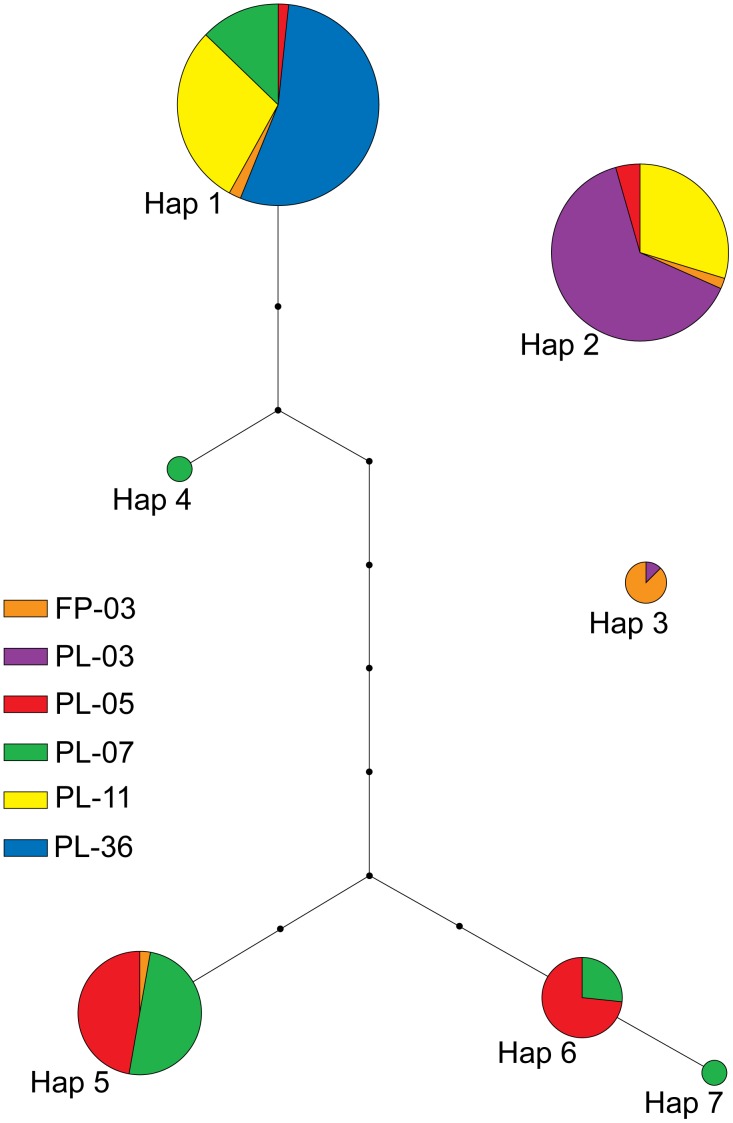
Statistical parsimony network of *T. newberryi* mtCR haplotypes. Each circle of the network represents a haplotype (Hap), the size of the circle is proportional to the number of individuals sequenced with that haplotype, each color represents a different sampled location, each line equates to one mutational step, and the small black circles are hypothetical haplotypes.

A TCS statistical parsimony network of *T. newberryi* mtCR sequences revealed that most individuals are of two haplotypes; 55 individuals had Haplotype 1 and 46 individuals had Haplotype 2 (Fig. 2). The placement of Haplotype 2 and 3 was not certain, but Haplotype 2 and 3 will connect to Haplotype 1 through an additional 12 and 16 mutational steps respectively, at 92% (data not shown). Two singleton haplotypes were observed from PL-07. The average distance between haplotypes was 6.6 mutational steps, excluding Haplotypes 2 and 3. In populations with more than one haplotype, the haplotypes were strongly divergent, with the average number of mutational steps between haplotypes greater than 6.6 steps, except for the singleton haplotypes in PL-07. For example, the 30 samples from PL-11 had two highly divergent haplotypes (with no connection at the 95% confidence level) indicating two genetically different maternal lineages within a population (12 mutational steps between Haplotypes 1 and 2 at 92%).

The overall Φ_ST_ value was highly significant (Φ_ST_ = 0.644, *P*<0.0033) indicating strong genetic differentiation across all populations of *T. newberryi*. Estimates of pairwise Φ_ST_ values indicated significant structure between sampled playas (*P*<0.0033, sequential Bonferroni correction), except for the comparison between PL-05 and PL-07 (Φ_ST_ = 0.059, *P* = 0.009) (Table 1). AMOVA indicated most of the variation was among populations (64.5%) versus within populations (35.5%) (Table S2 in File S1). Divergence between populations was not significantly associated with geographic distance (Mantel test, *P* = 0.65).

Of the 66 *T. l*. “short” samples sequenced, in contrast to *T. newberryi*, there was only one mtCR haplotype 549 bp in length (Genbank accession number KJ627792). The closest *T. newberryi* haplotypes to the *T. l*. “short” haplotype were Haplotypes 5 and 6, which differed by 18 pairwise differences. There were 18 polymorphic sites between the *T. l*. “short” haplotype and *T. newberryi* Haplotypes 5 and 6 consisting of 17 substitutions and 1 indel. No connection was found between the *T. l*. “short” haplotype and the *T. newberryi* haplotypes in the TCS network at any confidence level.

### Population Structure - microsatellites

Results of the AMOVA for *T. newberryi* indicated that 70.4% of the variation was within populations compared to 29.6% of the variation among populations (Table S2 in File S1). In contrast, the AMOVA for *T. l*. “short” indicated that 35.2% of the variation was within populations compared to 64.8% of the variation among populations (Table S2 in File S1). There was a significant difference (*P* = 0.001) when comparing the species overall *F*_ST_ values; *T. l*. “short” has a significantly greater degree of genetic structure and reduced gene flow than *T. newberryi*. The overall *F*_ST_ value for *T. newberryi* was 0.252 and every pairwise *F*_ST_ comparison was significant after Bonferroni correction (Table 2). The *F*_ST_ values ranged from 0.080 between FP-03 and PL-36 to 0.491 between PL-03 and PL-36. Pairwise comparisons between PL-03 and every other sampled playa consistently had the largest *F*_ST_ values in the dataset (*F*_ST_≥0.276; Table 2). The overall *F*_ST_ value for *T. l*. “short” was 0.600 and pairwise *F*_ST_ comparisons were significant after Bonferroni correction (Table 3) except for the comparison between PL-03 and PL-07. The *F*_ST_ values ranged from 0.024 between PL-07 and PL-08 to 0.794 between PL-03 and PL-33.

**Table 2:** *T. newberryi* mitochondrial control region Φ_ST_ values (below diagonal), microsatellite *F*_ST_ and *Nm* values in parentheses above diagonal.

Pop.	FP-03	PL-03	PL-05	PL-07	PL-11	PL-36
FP-03	––	0.276*(0.527)	0.188*(0.876)	0.092*(1.928)	0.200*(0.728)	0.081*(2.152)
PL-03	0.729*	––	0.488*(0.234)	0.356*(0.389)	0.468*(0.224)	0.491*(0.216)
PL-05	0.634*	0.813*	––	0.241*(0.668)	0.221*(0.706)	0.195*(0.856)
PL-07	0.617*	0.811*	0.059	––	0.215*(0.701)	0.154*(1.097)
PL-11	0.433*	0.479*	0.474*	0.415*	––	0.195*(0.755)
PL-36	0.846*	0.970*	0.765*	0.651*	0.448*	––

Asterisks indicate significance after Bonferroni correction (*P*<0.003).

**Table 3:** *T. l*. “short” microsatellite *F*_ST_ values (above diagonal) and *Nm* values (below diagonal).

Pop.	PL-03	PL-05	PL-07	PL-08	PL-09	PL-33
PL-03	––	0.657*	0.063	0.099*	0.422*	0.794*
PL-05	0.110	––	0.627*	0.678*	0.695*	0.261*
PL-07	2.889	0.126	––	0.024*	0.342*	0.720*
PL-08	1.660	0.095	7.168	––	0.398*	0.773*
PL-09	0.276	0.093	0.390	0.294	––	0.792*
PL-33	0.054	0.611	0.082	0.057	0.056	––

Asterisks indicate significance (*P*<0.003) after Bonferroni correction.

**Figure 3:**
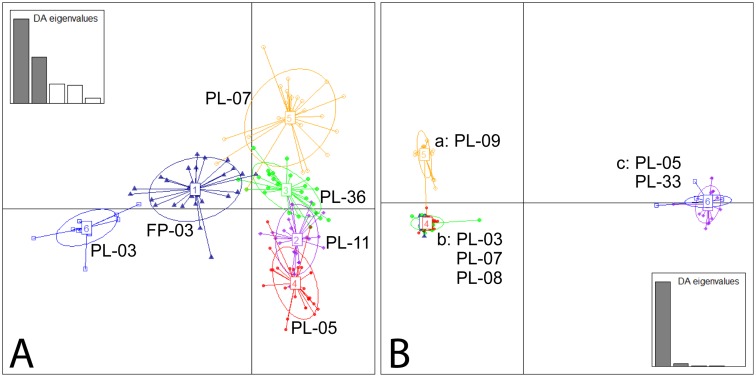
Discriminant analysis of principal components (DAPC) for *T. newberryi* (A) and *T. l*. “short” (B). The three clusters of the DAPC for *T. l*. “short” are designated as (a) PL-09, (b) PL-03, PL-07, PL-08 and (c) PL-05, PL-33. The insert graph displays the discriminant analysis eigenvalues with the largest two values in dark gray: *T. newberryi*, first eigenvalue was 287.2; the second eigenvalue was 157.8; *T. l*. “short”, first eigenvalue was 6167; the second eigenvalue was 219.9.

The shape of the DAPC scatterplot for *T. newberryi* was similar to that representing an island model of population structure, with four population clusters that overlapped (Fig. 3A [55]). The first axis of the DAPC separated the population ellipses consisting of individuals from PL-03 and FP-03 from the other populations (Fig. 3A). There were three distinct clusters in the DAPC scatterplot of *T. l*. “short” populations that were genetically more similar to each other than to the remaining populations [a (PL-09); b (PL-03, PL-07, PL-08); c (PL-05, PL-33); Fig. 3B]. Along the first axis in the DAPC for *T. l*. “short”, PL-05 and PL-33 were separated from the other populations (Fig. 3B). The second axis of the DAPC separated PL-09 from the remaining populations. The shape of the *T. l*. “short” scatterplot for the DAPC was similar to the population structure observed in a hierarchical island model [55], different from the island model seen in *T. newberryi*.

The *Nm* values between *T. newberryi* populations were generally below one, indicative of gene flow that is below the threshold for mitigating the effects of genetic drift [60] (Table 2). Despite assumptions that are likely violated in natural populations when calculating *Nm* [61], comparison of the relative degree of migration between species can still be made. Three pairwise comparisons had values that were slightly above one (*Nm* = 1.097 to 2.152): FP-03 vs. PL-07; FP-03 vs. PL-36; PL-07 vs. PL-36 (Table 2). The *Nm* values between *T. l*. “short” populations were below one, except for three pairwise comparisons (*Nm* = 1.660 to 7.168): PL-03 vs. PL-07; PL-03 vs. PL-08; PL-07 vs. PL-08 (Table 3).

### Genetic Diversity & Reproductive Mode

In *T. newberryi* populations, there were in total seven haplotypes, but the number of haplotypes in a population varied from one in PL-36 to five in PL-07. Although FP-03 had the least amount of sequenced individuals (*n* = 10), it had four haplotypes. The amount of nucleotide and haplotype diversity also varied from the lowest nucleotide and haplotype diversity in PL-36 at zero to the highest nucleotide diversity of 0.014 in FP-03 and the highest haplotype diversity in PL-07 at 0.625 (Table 1). For all populations combined, the nucleotide diversity was 0.015 and the haplotype diversity was 0.747. Modeltest results utilizing the AIC selected the TrN+I model as the best fit for the *T. newberryi* mtCR data. The smallest Tamura-Nei distance was between PL-05 and PL-07 (0.71%) and the largest occurred between PL-36 and FP-03 (2.34%) (Table S3 in File S1). The average genetic distance between playas was 1.60% and 1.79% based on p-distance and the Tamura-Nei model, respectively (Table S3 in File S1).

The number of alleles in *T. newberryi* microsatellite loci ranged from one to 14 with an average of 3.15 alleles across all loci (Table 1). The microsatellite TN-13, developed specifically for *T. newberryi*, was the most variable marker (21 alleles), while *T. l.* “short” specific markers were less variable (TLS-13, 2 alleles; TLS-5, 3 alleles; Table 1). The average allelic richness across all loci and populations was 4.12 (Table 1). The largest allelic richness was in PL-07 (4.36) and the smallest was in PL-11 (2.01). There was no evidence of linkage disequilibrium. Significant heterozygote deficiency after Bonferroni correction was observed for all loci in each population (Table 1).

For *T. l.* “short” the number of alleles ranged from one to seven with an average of 2.37 alleles across all loci (Table 1). Markers developed specifically for *T. l.* “short” (TLS-9 and TLS-5 [30]) were the most and least variable with nine and three total alleles, respectively. The average allelic richness across all loci and populations was 3.34 (Table 1) and was not significantly different to the allelic richness observed in *T. newberryi* populations (*P*>0.05). The largest allelic richness was in PL-07 (2.97) and the smallest was in PL-33 (1.54). Evidence of linkage disequilibrium was found in one playa lake (PL-09) only between loci TCB-99 and TLS-9. Observed heterozygosity was smaller than expected heterozygosity in each population and a significant departure (after Bonferroni correction) from Hardy-Weinberg expectations was observed in all populations (Table 1). The difference in observed heterozygosity between *T. l*. “short” and *T. newberryi* was not statistically significant (*P*>0.05).

In each population of *T. l.* “short” and *T. newberryi*, the individuals were examined for the presence or absence of a brood pouch, indicative of female or male. Every *T. l*. “short” individual had a brood pouch, congruent with the literature and the proposed parthenogenetic or hermaphroditic reproductive mode [27]. Also consistent with the prediction of *T. newberryi* as an androdioecious species [27], there were males observed within every *T. newberryi* population. The amount of males per population ranged from one male out of 30 samples (3.3% males) in PL-07 to eight males out of 28 (28.6%) in FP-03 (Table 1).

Inbreeding analysis indicated a significantly large overall *F*_IS_ value in *T. newberryi*: 0.601 (Table 1). All populations had large *F*_IS_ values, ranging from 0.394 in PL-11 to 0.889 in PL-03 (Table 1). The estimated proportion of selfing per population was smallest in PL-11 (*S* = 0.565), largest in PL-03 (*S* = 0.941) and a (geometric) mean for all *T. newberryi* populations was *S* = 0.740 (Table 1). There was no correlation between the values of *F*_IS_ or *S* in *T. newberryi* and the percentage of males per population. For example, PL-03 and PL-11 had the same percentage of males (13.3%), but the *F*_IS_ and *S* estimates for these populations were the largest and smallest observed, respectively. Like *T. newberryi*, inbreeding analysis in *T. l*. “short” indicated a significantly large *F*_IS_ value over all populations: 0.547 (Table 1). The population *F*_IS_ values for *T. l.* “short” ranged from 0.277 in PL-08 to 0.658 in PL-05. A statistical comparison of the overall *F*_IS_ values for the two species of *Triops* was non-significant (*P*>0.05). The proportion of overall selfing in *T. l*. “short” populations (geometric mean *S* = 0.689) was slightly lower than *T. newberryi*, with the largest proportion of selfing in PL-05 (*S* = 0.794) and the smallest in PL-08 (*S* = 0.433) (Table 1).

### Visual Assessment of Species Designation

**Figure 4:**
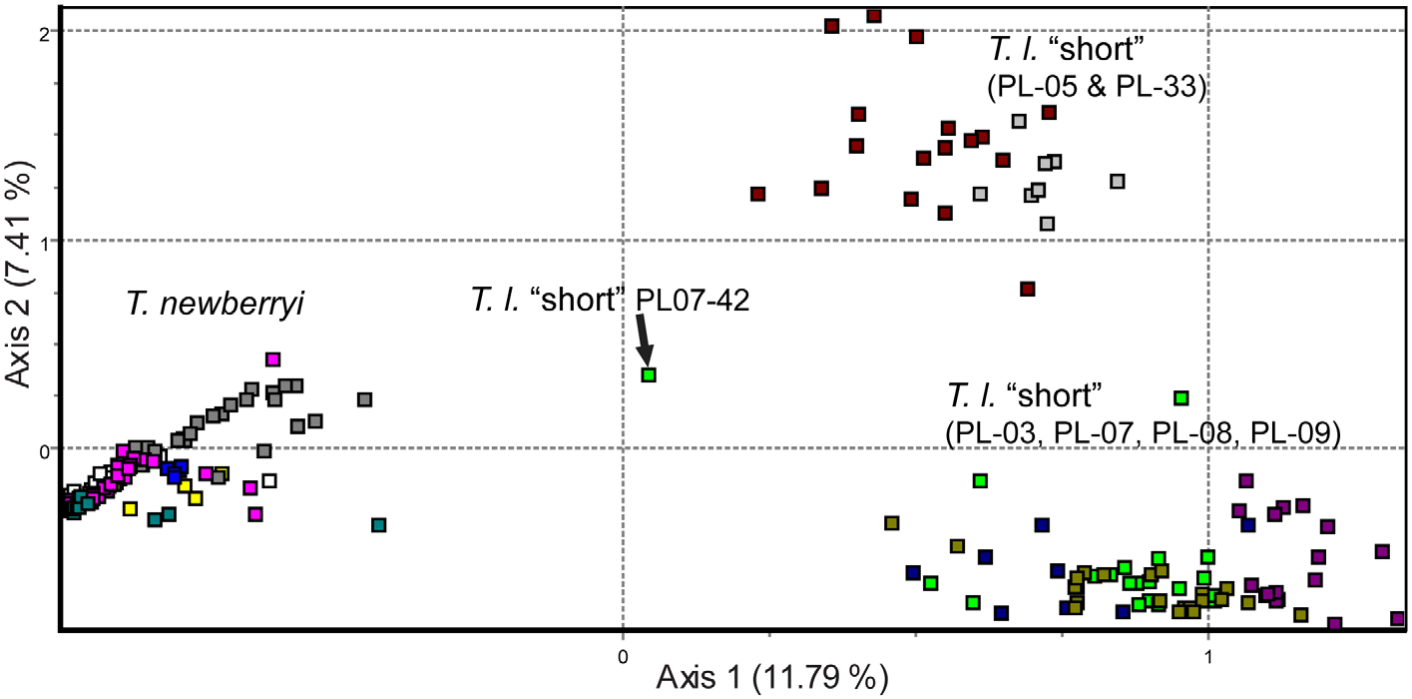
Factorial correspondence analysis (FCA) of *T. newberryi* and *T. l.* “short” populations combined. The first axis of the FCA represents 11.8% of the variation within the data and the second axis represents an additional 7.4%. Each colored square represents a different population and the population clusters are labeled by species on the graph.

The FCA separated *T. newberryi* populations from *T. l.* “short” populations, with the exception of one *T. l*. “short” individual that did not cluster with either species group (Fig. 4). There was one of the eight loci (TN-14) in this individual (PL07-42; Fig. 4) where the allele combination 157/161 was observed, but was not present in any other *T. l*. “short” at the same locus. The first axis of the FCA accounted for 11.8% of the variation within the data set and the second axis accounted for an additional 7.4% of the variation. Within *T. l*. “short”, there was a clustering pattern in the FCA similar to the DAPC, in which individuals from PL-05 and PL-33 separated along the second axis from the remaining four *T. l*. “short” populations (Fig. 4).

## Discussion

The results indicated that populations of *Triops* were highly structured genetically, even across short geographical distances. There was no evidence of a positive relationship between *F*_ST_ and geographic distance in either species. The genetic diversity varied across *Triops* populations, with slightly greater, but non-significant, overall genetic diversity in *T. newberryi* than in *T. l*. “short” for microsatellites and substantially higher haplotype diversity in the former species. Different models of population structure were observed for *T. newberryi* (island model) and *T. l*. “short” (hierarchical island) and both species of *Triops* had low estimated migration (*Nm*) between playa lake populations. Populations of *T. newberryi* and *T. l*. “short” were characterized by low diversity, a large degree of inbreeding and high proportions of selfing in all populations. There was no statistically significant difference between any of these measures (*F*_IS_, observed heterozygosity, allelic richness) between the species, but all interspecific differences except *F*_IS_ and the proportion of selfing (*S*) were consistent with population genetic theory. The presumed androdioecious species (*T. newberryi*) had higher diversity, fewer private alleles, higher heterozygosity, lower *F*_ST_, and greater within-population genetic variance. Lastly, there appears to be a clear genetic distinction between the putative species in southern New Mexico and no evidence of hybridization despite co-occurrence in some playa lakes.

### Population Genetic Structure

Despite the potential for dispersal across habitats located in close proximity, playa lake populations of *Triops* in this study have a high degree of genetic differentiation. For example, PL-03 and PL-05 are separated by a distance of only 1.45 km, however the microsatellite results indicate *T. newberryi* and *T. l.* “short” are each genetically distinct across the two ponds. There is no evidence of isolation by distance in either species with mtDNA or microsatellites, indicating factors beyond immigration and contemporary dispersal are influential in structuring *Triops* populations in southern New Mexico.

A survey of *T. cancriformis* in Europe [18] and of *Lepidurus packardi* in California [62] attributed the high genetic structure observed between populations to founder events and high selfing rates. Similarly in the current study, there is evidence that founder events and genetic drift, not contemporary gene flow, is responsible for the genetic structure of *T. newberryi* and *T. l*. “short” in southern New Mexico populations. The migration estimates between most populations are too small (<1) [60] to counteract the effects of genetic drift, suggesting that isolation and drift after the initial founding event is responsible for the genetic structure. There is an excess of private alleles (3–29%) in each species that can be due to populations that have been separated over time and have experienced little to no gene flow [63].

Despite both species being highly structured genetically, there was a significantly higher degree of differentiation (*F*_ST_ values) among the *T. l*. “short” populations. The difference is illustrated in the different shapes of the DAPC plots; the hierarchical island model as seen for *T. l*. “short” populations is congruent with increased differentiation as compared to the island model for *T. newberryi*. The differences in the *F*_ST_ values between species could be due to a variety of factors including influence from the mating system (discussed below), different population sizes, a genetic bottleneck, and/or distinct colonization events. For example, the *T. l*. “short” PL-05 and PL-33 populations cluster together in the DAPC scatterplot, possibly indicative of the same genetic lineage colonizing and successfully reproducing in both of these locations. In addition, *T. l*. “short” PL-03, PL-07, and PL-08 populations cluster together and have slightly increased *Nm* values (>1), which may indicate founding lineages and historical connectivity. Zierold et al. [31] demonstrated the possibility of long distance dispersal and range expansions within European populations of *T. cancriformis* after glacial maximum, but significant differentiation was still observed. This trend observed in populations of *Triops* is more consistent with founder events being relatively more influential for the genetic structure than contemporary dispersal.

### Comparison of Reproductive Mode

The highly inbred mating systems proposed for *Triops* species in southern New Mexico can also affect the genetic structure of populations. Populations of *T. l*. “short” in this study consist of all females (individuals have a brood pouch) that are thought to reproduce either through parthenogenesis or hermaphroditism [27], [32]. Androdioecy is the presumed reproductive mode for *T. newberryi* because there can be some proportion of “males” (individuals lacking a brood pouch) in a population and individuals with a brood pouch can reproduce in isolation [26], [27], [32]. In the current dataset, there was a ratio of “males” in *T. newberryi* populations ranging from 3.3%–28.6%. In comparing the results of the two *Triops* species with different presumed mating systems, *T. l*. “short” had fewer alleles, decreased allelic richness, and smaller H_E_ and H_O_ than *T. newberryi* (Table 1), however the comparison between species for these values were not statistically significant. Despite an absence of statistical significance, a selfing species, such as *T. l*. “short”, should have a lower number of alleles, reduced allelic richness and low observed heterozygosity [23]. Also consistent with the effect of mating systems between species is the increase in the greater number of private alleles in *T. l*. “short”, the hierarchical island model of population structure and larger between species variation in *T. l*. “short” when compared to *T. newberryi*.

Inconsistent with the theory for a selfing species is the inbreeding coefficient and proportion of selfing, as *T. newberryi* has slightly higher values than *T. l*. “short”, which should be lower if *T. newberryi* populations experience some benefit of outcrossing with androdioecious matings between hermaphrodites and males. The reduced microsatellite variation, greater population differentiation and the single mtCR haplotype for *T. l*. “short” that we observed could also be evidence for demographic differences between species including more population bottlenecks in *T. l*. “short” [64], that *T. l*. “short” is an evolutionarily more derived species than *T. newberryi* [65] or that the persistence of founder events is stronger within *T. l.* “short” populations. The differences in diversity between species could also be due to genotyping error or amplification success of microsatellite markers within a species, however, there is no indication that allele dropout with cross-species microsatellites is more prevalent in *T. newberryi* or *T. l*. “short” (results not shown). With mitochondrial genes, Vanschoenwinkel et al. [33] suggested close genetic affinity between *T. newberryi* and *T. l*. “short” and Macdonald et al. [32] reported only modest differences between *Triops* species. Despite some males within *T. newberryi* populations, the *F*_IS_ values were large for both species of *Triops* in southern New Mexico indicating an overall high degree of inbreeding. There was also no correlation between the fraction of males in each population and the *F*_IS_ values of *T. newberryi*, as would be expected if populations with more males experience more outcrossing, and therefore, a smaller inbreeding coefficient.

Using allozyme data, Sassaman et al. [27] concluded that there was a difference in genetic variation between self fertilizing *T. longicaudatus* populations and androdioecious populations of *T. newberryi* in the southwestern United States, but inbreeding values were not given. Zierold et al. [18] did not observe significant differences of *F*_IS_ values, heterozygosity, or allelic richness between *T. cancriformis* populations that had some males compared to populations with no males. Velonà et al. [21], however, did observe greater diversity in populations of *T. cancriformis* that contained males and are presumed to be outcrossing. While the genetic diversity may have differed between androdioecious and selfing populations, the inbreeding coefficient, as in this current study, was not able to distinguish between those populations that have some outcrossing compared to parthenogenetic populations [21].

Based on population genetic theory, there should be strong differences in the diversity and structure of a self fertilizing species compared to a species that experiences some outcrossing [23]. Our results suggest that there are genetic differences between the two species with different presumed mating systems, consistent with the predictions of population genetic theory, however, there was a lack of statistical significance when comparing *T. l*. “short” and *T. newberryi*. The aforementioned evolutionary history and/or demographic differences between the species may explain why *T. l*. “short” has decreased genetic diversity compared to *T. newberryi* despite both species experiencing the same level of inbreeding. The inbreeding coefficients and selfing rate estimates are inconsistent with what would be predicted if *T. newberryi* populations were composed of hermaphrodites that can outcross with males (androdioecy). This might also suggest that although *T. newberryi* has slightly greater genetic diversity, the role of androdioecy does not alleviate the effects of inbreeding with regard to loss of alleles with genetic drift. In a confirmed androdioecious clam shrimp [66], there was a linear decline in inbreeding as the proportion of males increased, but we did not observe this in our data. Our results call into questions the role of males in *T. newberryi*. If there is an insufficient level of outcrossing to decrease the estimate of inbreeding, then do males have a reproductive role within a population? We cannot exclude the possibility that individuals lacking a brood pouch are non-reproductive and do not contribute genetic material to subsequent generations. If this is true, then *T. newberryi* and *T. l*. “short” populations could be composed of hermaphrodites that can outcross with other hermaphrodites and both species could have the same mating system. Outcrossing in both species is supported by the large values of *F*_IS_ and selfing rates that are not near unity in value, which would indicate complete self fertilization as expected in a pure hermaphroditic scenario. Further clarification of the mating system will require a more direct method, such as a progeny array, to determine the reproductive mode of the *Triops* species within southern New Mexico. Work is also needed to clarify how adaptable the species are to their respective environments and if these differences contribute to the genetic diversity and structure of *T. newberryi* and *T. l.* “short” populations.

### Putative Species Designation

We presently have analyzed three ponds in which *T. l*. “short” and *T. newberryi* co-occur in Southern New Mexico in this study. In a global phylogeny of the Notostraca, Vanschoenwinkel et al. [33] concluded that *T. longicaudatus* “short” and *T. newberryi* were not monophyletic and should be considered conspecifics. It is of interest to know if the species hybridize when they co-occur or if they are reproductively isolated [27], [32]. The two species share a majority of their microsatellite alleles, although some alleles are more prevalent in one species.

The FCA of both species showed one individual from PL-07 that was ordinated in an intermediate position between the *T. l*. “short” and *T. newberryi* population clusters (Fig. 4); both species occur in PL-07. The morphology of this individual was consistent with *T. l*. “short” [32]. The alleles of one microsatellite (TN-14 locus, alleles 157/161) make this sample different. Lacking pedigree information, we are unable to determine if this condition is identical in state or identical by descent and thus a hybrid origin.

### Conclusion

The population genetic structure of *T. newberryi* and *T. l.* “short” in southern New Mexico appears to be strongly influenced by founder events and genetic drift. The high degree of genetic structure between populations at a local scale suggests that contemporary gene flow is not rapidly eroding persistent founder effects. There is evidence that *Triops* cysts may have the potential for dispersal via a variety of methods [1], [2], [3], [4], [5], [7], [8], but the results here suggest that the potential for dispersal is not realized either because immigration is too low to homogenize the genetic diversity or that migrants have a decreased ability to hatch and/or reproduce in ponds that are non-native. Despite slight differences in the amount of genetic diversity between the presumed selfing (*T. l*. “short”) and androdioecious species (*T. newberryi*), the results indicate similar levels of inbreeding occurring in both species. We conclude both species could be composed of hermaphrodites that can outcross with other hermaphrodites and androdioecy for *T. newberryi* remains unconfirmed. There are clear differences genetically between *T. l*. “short” and *T. newberryi* and little evidence to suggest hybridization between the species, which supports *T. l*. “short” and *T. newberryi* being distinct species as suggested by Macdonald et al. [32]. Additional work could further clarify the mating systems and determine adaptability of migrants to new habitats.

## Supporting Information

File S1 **Supporting Tables.** Table S1. GPS coordinates of each sampled playa location. Table S2. AMOVA results. Table S3. *Triops newberryi* mitochondrial control region genetic distances.
(DOCX)
