# Supplementary material for: Discriminating between the Effects of Founding Events and Reproductive Mode on the Genetic Structure of Triops Populations (Branchiopoda: Notostraca)
Source: PLoS One. 2014 May 13;9(5):e97473. doi: 10.1371/journal.pone.0097473 (PMC4019589; doi:10.1371/journal.pone.0097473)
Supplement: File S1 — Supporting Tables. Table S1. GPS coordinates of each sampled playa location. Table S2. AMOVA results. Table S3. Triops newberryi mitochondrial control region genetic distances. (DOCX) [file pone.0097473.s001.docx]

Table S1. GPS coordinates of each sampled playa location.

| Site | Latitude | Longitude |
| --- | --- | --- |
| FP-03 | N 32° 19' 13.7" | W 106° 44' 31.0" |
| PL-03 | N 32° 32' 23.5" | W 106° 53' 55.7" |
| PL-05 | N 32° 32' 25.3" | W 106° 53' 00.9" |
| PL-07 | N 32° 31' 54.1" | W 106° 47' 23.6" |
| PL-08 | N 32° 31' 38.0" | W 106° 46' 09.7" |
| PL-09 | N 32° 32' 41.2" | W 106° 54' 41.5" |
| PL-11 | N 32° 30' 31.6" | W 106° 44' 33.2" |
| PL-33 | N 32° 20' 03.9" | W 106° 59' 30.4" |
| PL-36 | N 32° 26' 12.2" | W 106° 44' 33.7" |

Table S2. AMOVA results for *T. newberryi* and *T. l*. “short” with the mtCR and the microsatellite data where D. F. is the degrees of freedom.

|  | **Source of Variation** | **D.F.** | **Sum of Squares** | **Variance** | **Percent Variation** |
| --- | --- | --- | --- | --- | --- |
| ***T. newberryi* mtCR** | Among Populations | 5 | 410.18 | 3.06 | 64.46 |
|  | Within Populations | 154 | 259.87 | 1.69 | 35.54 |
| ***T. newberryi* msats** | Among Populations | 5 | 271.07 | 1.84 | 29.60 |
|  | Within Populations | 157 | 687.87 | 4.38 | 70.40 |
| ***T. l*. "short" msats** | Among Populations | 5 | 706.00 | 5.37 | 64.83 |
|  | Within Populations | 150 | 437.19 | 2.91 | 35.17 |

Table S3. *T. newberryi* mitochondrial control region genetic distances. Tamura-Nei model of diversity is below the diagonal; p-distance is above the diagonal.

|  | **FP-03** | **PL-03** | **PL-05** | **PL-07** | **PL-11** | **PL-36** |
| --- | --- | --- | --- | --- | --- | --- |
| **FP-03** | - | 1.79% | 1.23% | 2.17% | 2.06% | 2.27% |
| **PL-03** | 1.85% | - | 1.91% | 2.08% | 1.21% | 2.21% |
| **PL-05** | 2.21% | 1.95% | - | 0.70% | 1.57% | 1.30% |
| **PL-07** | 2.25% | 2.13% | 0.71% | - | 1.47% | 0.96% |
| **PL-11** | 2.13% | 1.24% | 1.60% | 1.51% | - | 1.02% |
| **PL-36** | 2.34% | 2.26% | 1.32% | 0.97% | 1.05% | - |
